# Sigmoid and Transverse Sinus Thrombosis as a Complication of Chronic Suppurative Otitis Media in a Child: A Case Report and Review of Literature

**DOI:** 10.1002/ccr3.71720

**Published:** 2025-12-30

**Authors:** Abdullah Nadeem, Hafiza Hafsa, Ruqaiya Shahid Raja, Mahad Shahid Raja, Muhammad Saad Abbas, Omer Javed Khan, Faisal Khalique, Riyan Imtiaz Karamat, Aymar Akilimali

**Affiliations:** ^1^ Department of Medicine Dow University of Health Sciences Karachi Pakistan; ^2^ Dow Medical College Karachi Pakistan; ^3^ Shifa College of Medicine Islamabad Pakistan; ^4^ Akhtar Saeed Medical College Lahore Pakistan; ^5^ Aga Khan Medical College Karachi Pakistan; ^6^ Central Park Medical College Lahore Pakistan; ^7^ Lahore Medical and Dental College Lahore Pakistan; ^8^ Rahbar Medical and Dental College Lahore Pakistan; ^9^ Department of Research, Medical Research Circle (MedReC) Goma Democratic Republic of Congo

**Keywords:** antibiotic, anticoagulation management, chronic suppurative otitis media, complications, sigmoid sinus thrombosis

## Abstract

Chronic suppurative otitis media is an easily managed condition with potential for severe sinus thrombophlebitis complications secondary to cholesteatoma, if not diagnosed and treated promptly. Diagnosis relies on the culture and sensitivity of the drained suppuration, computed tomography (CT), and magnetic resonance imaging (MRI). Treatment entails a combination of all or some surgical, antibiotic, and anticoagulant use with postoperative follow‐up. Our 8‐year‐old female patient presented with chronic left postauricular swelling with foul‐smelling otorrhea and associated otalgia. More recently, she developed fever, headache, and vomiting. Fundoscopy noted papilledema. After ear examination, emergency incision and drainage were conducted with the suppuration sent for culture analysis. Tuberculosis was also ruled out. The patient had no history of dental caries. Contrast CT scan of the brain revealed features suggestive of partial thrombosis of the superior sagittal, left transverse sinus and sigmoid sinus extending up to the jugular vein. MRI and magnetic resonance venography (MRV) also indicated left otomastoiditis. Surgical treatment involved left‐sided tympanomastoidectomy and otomastoidectomy. Anticoagulant Rivaroxaban was used 15 mg twice daily for 21 days. Postoperatively, the dosage was increased to 20 mg once daily for 3 months. Antibiotic vancomycin was administered preoperatively. Postoperative CT showed good prognosis. Postoperatively there was gradual improvement in headache and vision deterioration of right eye; fundoscopy showed improvement in papilledema, patient leukocyte counts also declined significantly, eventually to 13.4/μL. Anticoagulant and antibiotic therapy was continued. Some authors advocate only blood–brain barrier crossing antibiotics, while others employ surgical treatment. The risks involved with using anticoagulants must be carefully weighed before use. Our case presents classic clinical signs and symptoms.

## Key Clinical Message

Chronic suppurative otitis media can lead to life‐threatening sinus thrombosis; early imaging, surgical drainage, and prompt antibiotic‐anticoagulant therapy are essential for preventing neurological complications and ensuring recovery. Chronic suppurative otitis media can lead to life‐threatening sinus thrombosis; early imaging, surgical drainage, and prompt antibiotic‐anticoagulant therapy are essential.

## Graphical abstract

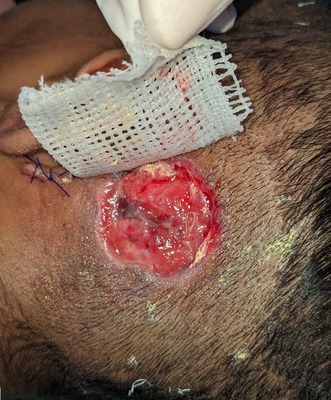

**Abbreviations**
AFB: acid‐fast bacilli
CSOM: chronic suppurative otitis media
CT: computed tomography
ENT‐HN: ear, nose, throat‐head and neck
MRI: magnetic resonance imaging
MRV: magnetic resonance venography

## 1 Introduction

Chronic suppurative otitis media (CSOM) is a major cause of preventable hearing loss, especially in socioeconomically deprived populations [1]. It takes place when tympanic membrane perforation leads to persistent middle ear inflammation, hearing loss, and otorrhoea. Topical antibiotics are the primary treatment, while surgery may be needed for persistent cholesteatoma to repair the tympanic membrane and improve hearing [1]. Cholesteatoma, a complication of CSOM, can lead to bony sinus wall erosion leading to thrombosis [2]. Thrombophlebitis of the dural venous sinuses is a rare complication of otitis media, often with sepsis [2]. Diagnosis relies on clinical signs, imaging such as CT and MRI, with the delta sign on CT and lab tests [2]. Surgical treatment, including ear surgery and lateral sinus exposure, is needed for septic cases. Prognosis is good if no other intracranial complications are present [2]. A delay in the recognition of intracranial complications in children and in the application of appropriate therapy may result in morbidity and mortality [3]. Children with recurrent fever, headache, and a recent history of acute and chronic otitis media should be evaluated for the possibility of sigmoid sinus thrombophlebitis [4]. Early diagnosis and surgery is necessary for a better prognosis in sigmoid sinus thrombophlebitis, and hence, radical mastoidectomy combined with anti‐infection therapy is the main treatment alongside anticoagulation therapy [5]. The clinical diagnosis of cerebral venous thrombosis is rarely suspected before a CT scan and MRI [6]. Most physicians opted for a CT scan as the primary imaging modality to diagnose thrombophlebitis. The most common findings found on CT scan are the triangle sign and empty delta sign; on MRI, a loss of flow void is frequently present [6]. Our case report presents our viewpoint on the diagnosis and prompt treatment of CSOM alongside the potentially fatal complications such as cerebral sinus thrombophlebitis.

## 2 Case Presentation

### 2.1 Case History/Examination

We report the case of an 8‐year‐old girl from rural Sindh, Pakistan. She is developmentally healthy and fully immunized according to the National Immunization Program. She presented to the pediatric emergency department of a tertiary care hospital in Karachi for surgical evaluation. Her symptoms include swelling behind the left ear for the past one and a half months, gradually accompanied by pain and discharge. The discharge was scanty, foul‐smelling, non‐bloody, and greenish in color, *exuding* from the left ear. In addition, she has been experiencing fever, vomiting, and headache for 2 weeks.

In the month prior to her admission, her main complaint was swelling and tenderness behind her left ear. The swollen area was painful to touch and showed redness on the skin above it. On arrival at our institute, she presented with a high‐grade fever that had persisted for the past 2 weeks, with two to three episodes daily, lasting 2–3 h. The fever was accompanied by chills, excessive sweating followed by a drop in temperature. The fever was abrupt in onset, was continuous and peaked at 103°F. It was temporarily alleviated by medication. Fever was associated with a headache in the fronto‐parietal region, which also had a sudden onset and was of moderate intensity. About 10 days after the onset of fever, she developed vomiting, which was non‐projectile, non‐bilious, non‐bloody, and contained food particles. She also reported right jaw and neck pain for the last 15 days. Additionally, the purulent discharge from her left ear had worsened in the previous week. There is no history of prior tooth extractions, nor any reports of tinnitus, ear fullness, vertigo, ataxia, loss of consciousness, abnormal body movements, hematuria, hematemesis, or similar conditions in her medical history. Her medication history in the past month only included analgesics and antipyretics.

On examination, she appeared ill, displayed tachycardia (pulse rate: 140 bpm), normal blood pressure, normal respiratory rate (20 cycles/min), she was febrile with a temperature of 101.8°F and 98% oxygen saturation. There was no evidence of pallor, cervical lymphadenopathy, or pedal edema. Examination of the left ear revealed a painful, fluctuant postauricular swelling that discharged foul‐smelling green pus upon spontaneous rupture. The external auditory canal was also filled with pus. An emergency postauricular incision and drainage procedure was performed, collecting pus for culture, which tested positive for *Pseudomonas Aeruginosa* . Laboratory tests at presentation indicated progressive leukocytosis starting at 11.3, increasing to 13.9, and ultimately reaching 24.7/μL, along with anemia (8.9 g/dL) and hypoalbuminemia (2.67 g/dL). Pus samples were also tested with Gene Xpert and acid‐fast bacillus smear to rule out tuberculosis, which is endemic in Pakistan, and the results returned negative. Blood culture showed no growth. The Infectious Disease team was consulted and recommended intravenous ceftriaxone and vancomycin. It is to be noted that the patient belongs to a rural area with no advanced medical treatment and high illiteracy which may have led to the advanced disease progression observed.

### 2.2 Imaging Investigation Findings

**FIGURE 1:**
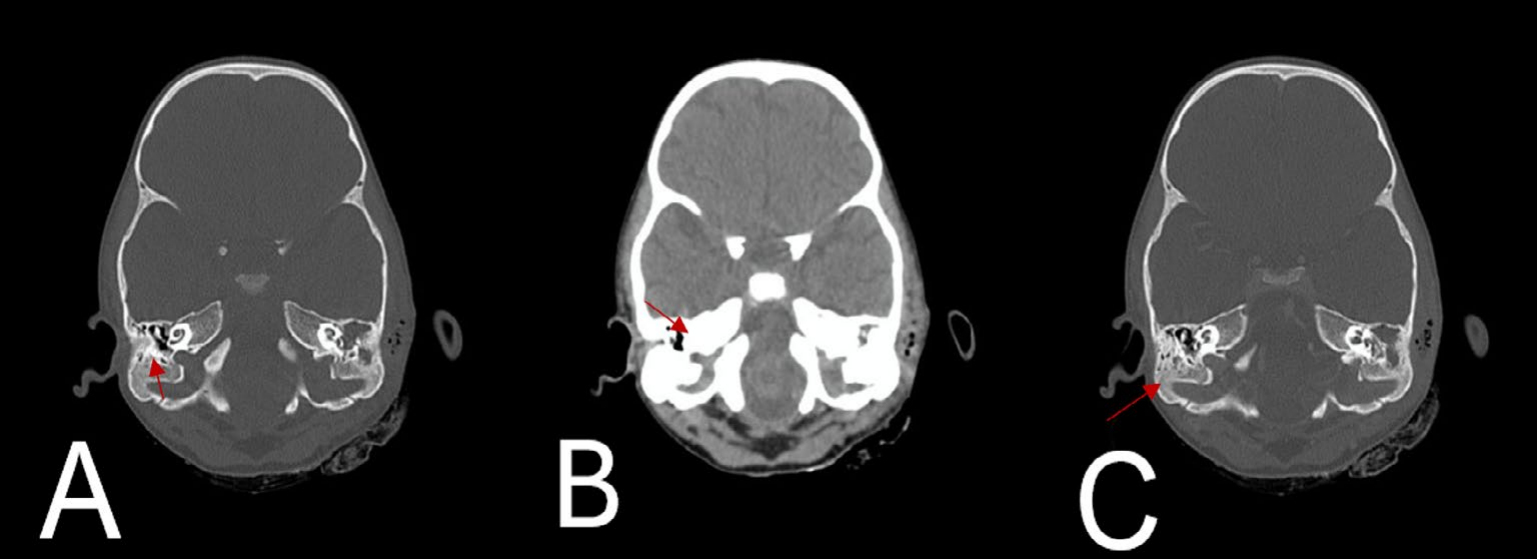
(A) Contrast‐enhanced CT scan of the brain showing the right mastoid air cells (arrow) show sclerosis and partial opacification consistent with chronic mastoiditis. (B) A slightly anterior section demonstrates soft‐tissue density opacification within the right middle ear cavity (arrow), involving the epitympanum and mesotympanum, indicating otitis media with effusion or granulation tissue formation. (C) A more posterior image shows residual fluid density within the right mastoid tip (arrow) without bony destruction or intracranial extension. The left mastoid and middle ear are well aerated in all sections.

The child reported difficulty in head movement, experiencing increased pain when tilting it forward and to the left. A contrast CT scan of the brain revealed findings indicative of acute on chronic oto‐mastoiditis, showing an abscess in the pre‐mastoid space (Figure 1A) and thrombosis in the left transverse and sigmoid sinuses, extending to the jugular vein (Figure 1B). Furthermore, there was evidence of partial opacification in the right mastoid air cells (Figure 1C), suggesting acute on chronic oto‐mastoiditis with abscess formation in both the pre‐mastoid and post‐mastoid spaces.

A neurological consultation was conducted based on the CT scan findings. The examination revealed a visual acuity of 6 × 6 in the left eye, while the right eye had decreased acuity, allowing the patient to read from a distance of 1 m. No gross motor or sensory loss was noted. Fundoscopy showed papilledema in both eyes, significantly more pronounced in the right eye. Following an in‐depth discussion with neurologists and neurosurgeons, Rivaroxaban was prescribed; this factor Xa inhibitor is used for treating deep vein thrombosis (DVT) and pulmonary embolism (PE). The initial dosage was set at 15 mg twice daily for 21 days, subsequently increasing to 20 mg once daily for 3 months.

**FIGURE 2:**
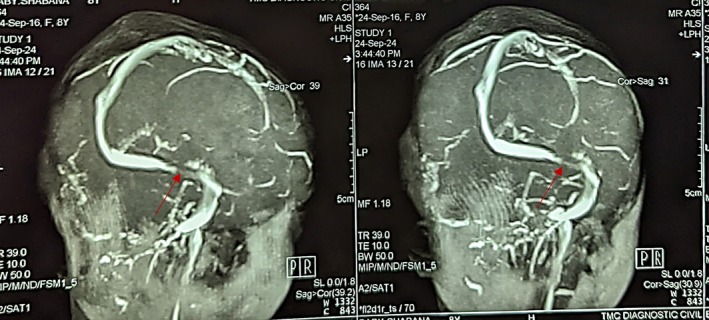
MRV showing filling defect in the left sigmoid sinus (arrow) and evidence of left otomastoiditis with right subgaleal hematoma.

**FIGURE 3:**
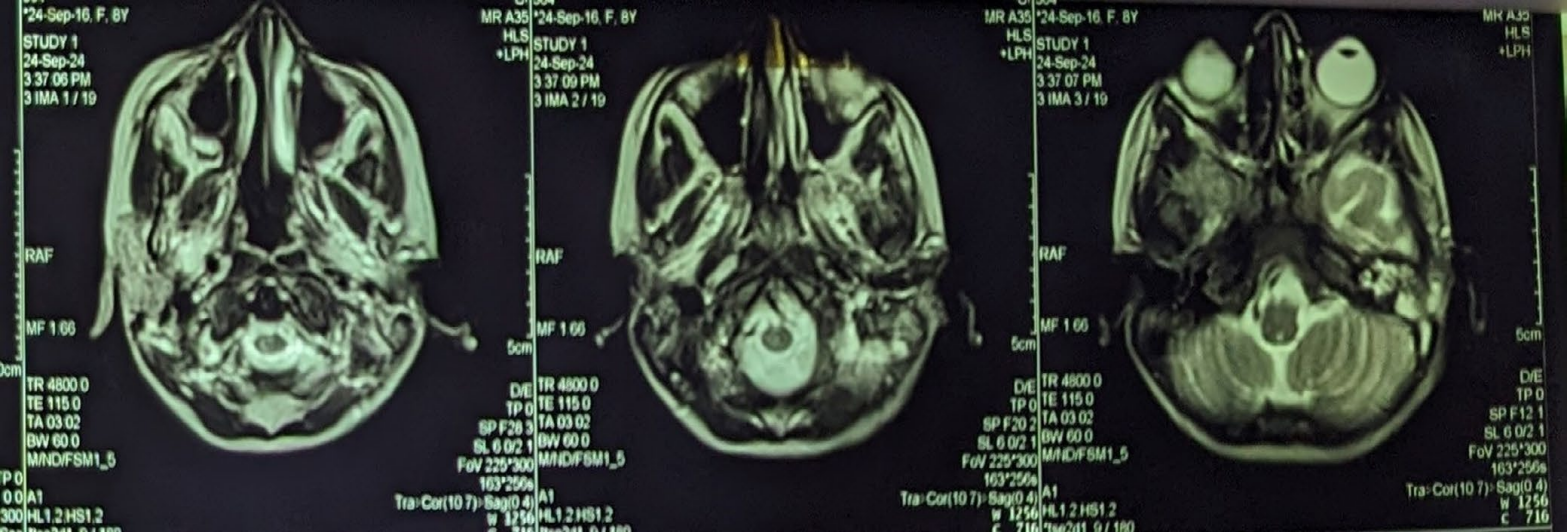
MRI showing loss of flow void in the left transverse and sigmoid sinuses, suggestive of thrombosis.

MRI and MRV revealed abnormal signals in the left mastoid sinus, extending to the left external ear and the area in front of the ear (Figure 2). Loss of flow void was observed in the left transverse sinus and left sigmoid sinus, with a noted filling defect during post‐contrast enhancement (Figure 3). These findings indicated left otomastoiditis that extends to the left external ear, alongside a right subgaleal hematoma, with no signs of intracranial involvement.

### 2.3 Surgical Management

Due to a collection of pus in the postauricular region, an emergency incision and drainage was performed via a postauricular incision in the ENT Emergency Department. Given the unavailability of resources or emergent clinical need for definitive bone work at that time, a complete mastoidectomy was not performed during the initial emergency procedure. Post incision and drainage, the patient was initiated on a course of systemic antibiotics (which, in the full case, included Ceftriaxone and Vancomycin). Despite 15 days of antibiotic therapy in the ward, the patient showed no signs of satisfactory clinical improvement, prompting the need for definitive surgical intervention. Consequently, an elective mastoidectomy (specifically, a left‐sided tympanomastoidectomy in the full case) was performed 15 days following the initial treatment to address the underlying chronic Otomastoiditis and associated complications.

Fifteen days after admission, the patient underwent a left‐sided tympanomastoidectomy. Postauricular incision was given, and temporal fascia was unhealthy looking. A periosteal elevator was used to expose the margins of the MacEwen's triangle. Otomastoidectomy had already been done by the subperiosteal abscess.

**FIGURE 4:**
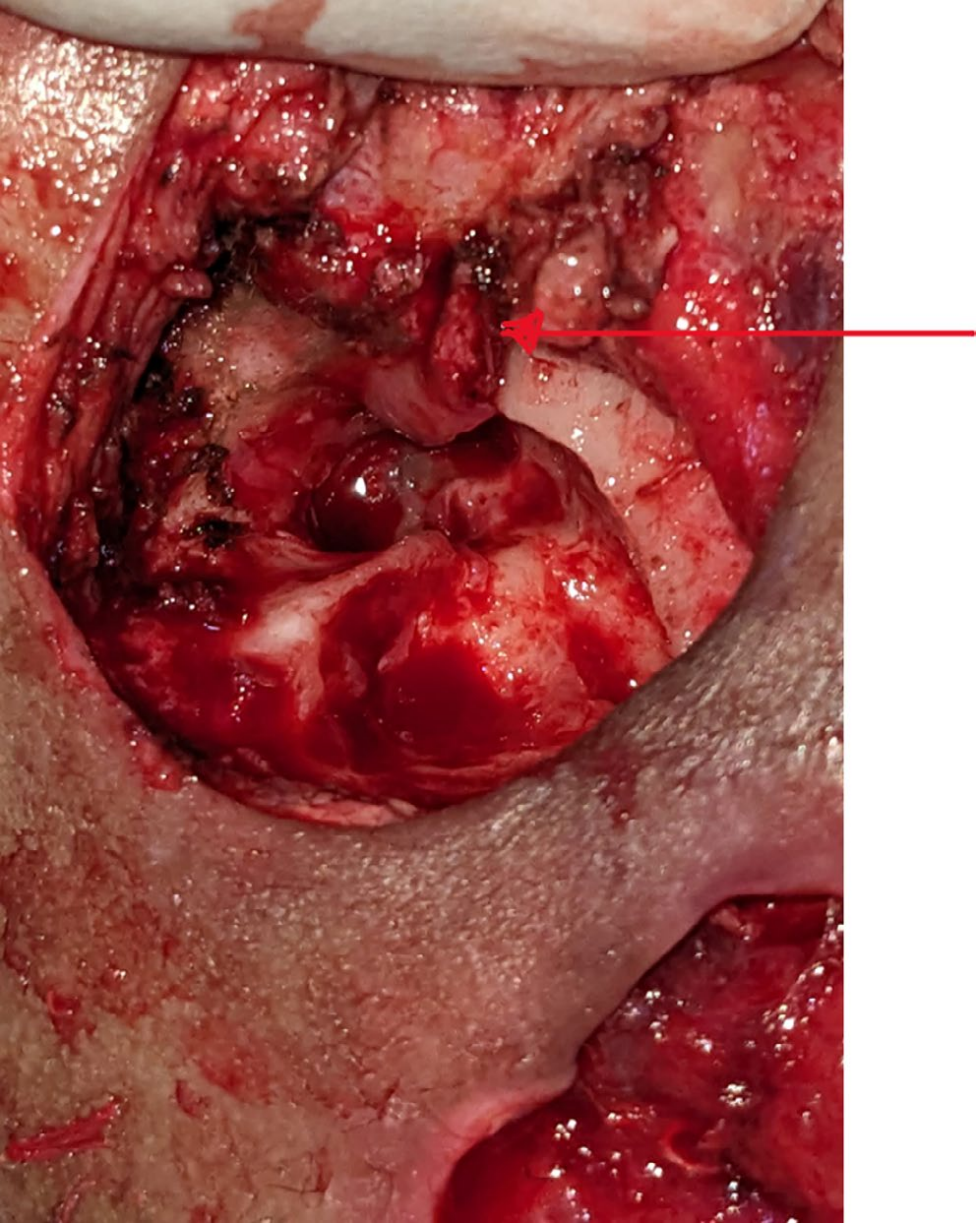
Perioperative finding of granulation tissue in attic and middle ear; canal wall down mastoid cavity after clearance.

**FIGURE 5:**
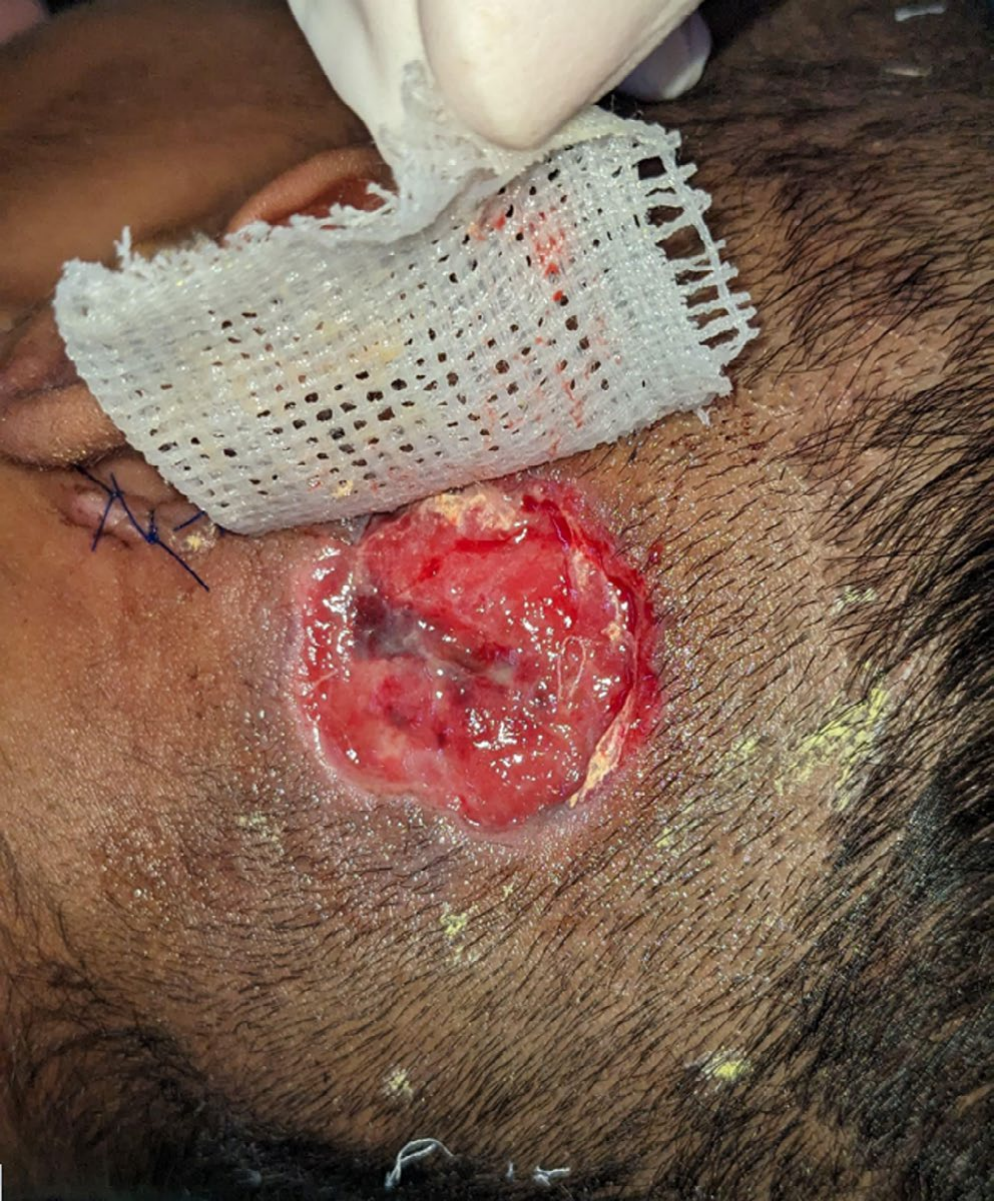
Postoperative day 10: Healed incision site after suture and pack removal.

Granulation tissue was removed from both the attic and middle ear cavity, with the incus involved in the disease process; however, the other ossicles were not identified (Figure 4). The canal wall was down, and the entire cavity was cleared with meatoplasty performed. The surgical team did not remove the thrombotic clot from the sigmoid/transverse sinus; the purpose of the mastoidectomy was to clear the source of infection. The patient was concurrently kept on Rivaroxaban anticoagulant therapy for the purpose of managing the sinus thrombosis. The postoperative period progressed without complications, and rivaroxaban was continued alongside ceftriaxone as the prescribed antibiotics. On the tenth postoperative day, the suture and packing were removed, as illustrated in Figure 5.

### 2.4 Follow‐Up Management and Investigations

The patient showed improved outcomes after surgery, with gradual improvements in headaches and vision deterioration of the right eye. Fundoscopy also revealed improvement in previously identified edema, and the patient's leukocyte counts declined significantly from 24.7 to 15.5, and eventually to 13.4/μL. Tables 1 and 2 show a summary of the entire management alongside detailed investigations and labs of the patient.

**TABLE 1:** Detailed timeline of clinical events, diagnostics, and interventions.

Date/day relative to admission	Event/intervention	Details
~45 days before admission	Onset of initial symptoms	Left postauricular swelling, mild otalgia, scanty green foul‐smelling otorrhea. No neurological signs.
~15 days before admission	Systemic symptoms began	High‐grade fever (up to 103°F), 2–3 episodes/day with chills and sweating, responsive to antipyretics.
~10 days before admission	New symptoms	Vomiting (non‐projectile, non‐bloody), headache localized to the frontoparietal region, pain on right jaw and neck movement.
Day 0: Admission	Emergency presentation to ENT‐HN unit	Ill‐looking, febrile (101.8°F), tachycardic (140 bpm), normal BP and oxygen saturation. Tender, fluctuant swelling behind the left ear with spontaneous discharge.
Day 0	Initial procedures and labs	Emergency incision and drainage; pus collected and sent for culture and GeneXpert/AFB.
Day 1–2	Imaging: Contrast‐enhanced CT	Revealed left otomastoiditis, abscess in pre‐ and post‐mastoid spaces, and thrombosis of left transverse/sigmoid sinus extending to the jugular vein (Figure 1).
Day 3	Neurological evaluation and fundoscopy	Papilledema in both eyes (worse in right); decreased vision in right eye; no motor or sensory loss.
Day 3–4	Start of anticoagulant therapy	Rivaroxaban initiated: 15 mg BID for 21 days.
Day 5	Imaging: MRI and MRV	Confirmed loss of flow void in left transverse/sigmoid sinuses, filling defect with post‐contrast enhancement, right subgaleal hematoma (Figures 2 and 3).
Day 5	Infectious disease consultation	Blood culture: no growth. GeneXpert & AFB smear: negative. Antibiotics prescribed: IV ceftriaxone and vancomycin.
Day 15	Definitive surgical intervention	Left tympanomastoidectomy with granulation clearance, incus involvement noted; canal wall down, meatoplasty done (Figures 4 and 5).
Post‐op Day 10	Follow‐up and recovery	Suture and pack removed. Improved headache and right eye vision. WBC trending down. Fundoscopy: reduced papilledema.
Post‐op Weeks 2–6	Continued recovery	Rivaroxaban 20 mg once daily continued for 3 months. Stable vitals, healing surgical site, and resolving leukocytosis.

**TABLE 2:** Laboratory and microbiological investigations.

Investigation	Result/value	Date/timing	Reference range/interpretation
White blood cell count (WBC)	11.3 → 13.9 → **24.7 × 10** ^**9**^ **/L** → 15.5 → 13.4	Day 0 to Post‐op Week 2	Elevated throughout admission; indicates ongoing infection; trended down with therapy.
Hemoglobin (Hb)	**8.9 g/dL**	Day 0	Low—indicates anemia, possibly secondary to chronic disease or infection.
Serum albumin	**2.67 g/dL**	Day 0	Low—suggests chronic inflammation or poor nutritional status.
Pus culture	*Pseudomonas aeruginosa*	Day 1	Confirmed bacterial etiology of otomastoiditis and abscess.
GeneXpert (TB PCR)	Negative	Day 1–2	Tuberculosis ruled out.
AFB smear	Negative	Day 1–2	No acid‐fast bacilli seen.
Blood culture	No growth	Day 1–2	No systemic bacteremia at presentation.
Imaging (CT brain with contrast)	Otomastoiditis, abscess, and sinus thrombosis	Day 2	Features of chronic infection with vascular involvement (Figure 1).
MRI + MRV	Loss of flow void, sinus thrombosis, subgaleal hematoma	Day 5	Confirmed diagnosis of dural venous sinus thrombosis with no intracranial extension (Figures 2 and 3).

*Note:* Bold values indicate progressive leukocytosis starting at 11.3, increasing to 13.9, and ultimately reaching 24.7/μL, along with anemia (8.9 g/dL) and hypoalbuminemia (2.67 g/dL).

## 3 Discussion

Chronic suppurative otitis media is a common occurrence among people and careful consideration should be given to treat it, or else severe complications can develop. Even though the introduction of antibiotics has decreased the incidence of these complications, they have not been eliminated [7]. In this instance, the patient was 8 years old. This is consistent with the literature, which reports that patients with CSOM experience problems at any age between 4 and 72, with a typical age of 15–24 years [7]. A mural thrombus forms when fibrin, red blood cells, and platelets stick to the tissue. This thrombus may extend to the subcutaneous tissue, the jugular vein bulb, and other locations. It may also give rise to an emboli [8, 9, 10]. The temporal bone is next to the transverse and sigmoid sinuses. An inflammatory process gives rise to a phlebitis area and thrombus, the inflammation is due to spread by tributary veins or erosion via the temporal bone directly [9].

Sigmoid sinus thrombosis is a rare condition that can be brought on by infections, thrombophilia, head trauma, some cancers, and intravenous drug use. Symptoms can range from increased intracranial pressure, fever, otalgia, headache, vomiting, cranial nerve palsies, papilledema, altered mental status, seizures, stupor, and coma [11, 12, 13, 14, 15, 16]. Infection reaches the sigmoid sinus by either a middle ear infection or directly through the mastoid emissary vein, firstly it causes the production of perivascular microabscesses before further spreading, this affects the venous system, and creates an infected thrombus [17].

There are two ways in which a cholesteatoma causes a sigmoid sinus thrombophlebitis: a cholesteatoma directly erodes the bone plate of the wall of the sigmoid sinus causing compressive necrosis of intima; also, inflammatory lesions extend from veins in the mastoid cavity to the sigmoid sinus, leading to thrombus formation [18]. The most frequent clinical features seen in sigmoid sinus septic thrombophlebitis are fever, otalgia, otorrhea, and postauricular edema [14]. Headache is a very common neurological symptom seen in these cases, which was evident in our patient, and also seen in the cases in our literature review table [8, 11, 13, 15] (Table 3). Fever and otorrhea are the features consistently seen in all the studies [8, 9, 10, 11, 12, 13, 14, 15, 16], including ours.

**TABLE 3:** Diagnosis and management of past sinus thrombosis case reports secondary to CSOM.

Author (year)	Age/gender	Symptoms	Location of thrombophlebitis	Additional complications	Diagnostic aids used	Management plan	Recurrence
Kinal et al. (1958) [8]	7/Male	Right ear otorrhea with reduced hearing, bi‐frontal headache, anorexia, vomiting	Right lateral sinus	—	Sinography	Mastoidectomy, antibiotics	—
Neto et al. (1998) [9]	7/Male	Bilateral otorrhea and hearing loss, vomiting, fever and right cervical swelling	Right lateral sinus	Cervical abscess	CT	Radical mastoidectomy, antibiotics	None seen at 11 month follow up
Miura et al. (2005) [10]	15/Male	Hearing loss, otorrhea, otalgia, neck rigidity, diplopia, blurred vision	Lateral sinus	Meningitis	CT, MRI	Radical mastoidectomy and antibiotics	—
Zinis et al. (2006) [11]	5/Male	Fever, otalgia, otorrhea, headache, torticollis, vomiting, cervical swelling.	Proximal part of internal jugular vein	Factor (V) Leiden deficiency	CT, USG	Simple Mastoidectomy and a myringotomy	30 months post‐op there were no episodes of otitis media or venous thrombosis
Zinis et al. (2006) [11]	7/Male	Otorrhea, fever, headache, torticollis, neck swelling	Proximal portion of the internal jugular vein involving the jugular bulb	—	CT, MRI, MRV	Mastoidectomy with posterior tympanotomy	2 years post‐op no new episodes
Saha et al. (2007) [12]	11/Male	Fever, right sided torticollis, right ear purulent otorrhea	Right internal jugular vein, right sigmoid sinus	Otogenic lung abscess	CT, USG	Radical mastoidectomy, thromboembolectomy and antibiotic therapy for lung abscess	—
Viswanatha et al. (2011) [13]	12/Female	Fever, vomiting, headache, right purulent otorrhea	Right lateral sinus	Brain abscess	CT, MRI, MRV	Radical Mastoidectomy, drainage of brain abscess	Unremarkable at 6 month follow up
Ebrahimpour et al. (2020) [14]	29/Male	Fever, otalgia, right otorrhea, tinnitus and vertigo, right neck swelling	Right sigmoid sinus	Mastoid abscess	CT, MRI	FNA with USG guide for mastoid abscess and antibiotic therapy	—
Anqi et al. (2022) [15]	39/Male	Fever, chills, headache, neck swelling, left sided otorrhea, left ear hearing loss	Left sigmoid sinus and transverse sinus	Bezold abscess	CT	Mastoidectomy and abscess drainage with antibiotic therapy	2 month follow up unremarkable
Shah et al. (2023) [16]	9/Female	Fever, right otorrhea, right sided neck pain and vomiting	Right sigmoid sinus, transverse sinus, internal jugular vein	—	CT	Antibiotics	There was symptomatic improvement after a couple of weeks
Our case	8/Female	Fever, left otorrhea, headache, vomiting, neck pain	Superior sagittal, left transverse sinus, sigmoid sinus, internal jugular vein	—	CT, MRI, MRV	Mastoidectomy, antibiotics, anticoagulants	—

The most radiological studies used for diagnosis are CT and MRI. ENT specialists most often utilize a CT scan without contrast for evaluating patients with chronic otitis media; they deem it an efficient way to observe changes in bone, but it is not very sensitive to detect thrombosis [19]. CT was the primary modality used in most of the cases in the literature reviewed [9, 10, 11, 12, 13, 14, 15, 16], including our case (Table 3). A CT scan with contrast should always be ordered in cases of otitis media problems because the “empty triangle” image in the sigmoid sinus area, encircled by dura contrast, also known as the “empty delta sign,” is thought to be a common indication of thrombosis [20]. Although the author Kinal et al. used a sagittal sinogram to diagnose the site and extent of the thrombophlebitis [8].

Due to the high probability of multiple complications in a patient with CSOM, CT and MRI are both recommended [7]. A loss flow void or slow flow on MRI indicates thrombosis [11], a loss flow void was also seen in our patient's MRI. Our patient, including the case seen in this literature review, also underwent an additional diagnostic test, called an MRV [13]. The main diagnostic test of choice and follow up of cerebral venous thrombosis is an MR venography; it has many pros as it is noninvasive, can be done at the same time as an MRI, and does not incorporate ionizing radiation [11]. Garcia et al. also mention in a retrospective study that the MRI indicators of sinus thrombosis include high signal intensity on T, T‐weighted images, and absence of flow within thrombosed sinuses on gradient echo images. The proximity to dense bony structures causes artifacts, due to which the CT is labeled as less sensitive [20]. This artifact is attributed to partial volume averaging [21]. This claim is seconded by Saha et al. that MRI is more sensitive to detect thrombosis [12].

There were no additional complications seen in our case, however many of the cases in our reviewed cases included additional complications [9, 10, 11, 12, 13, 14, 15]. A brain abscess [13] is the second most intracranial complication of otitis media after meningitis [10]. An otogenic brain abscess is found most commonly in the cerebellum and the temporal lobe [13]. Anqi et al. reports a bezold abscess in their patient, it is mostly caused by Gram‐negative bacillus, *Staphylococcus aureus* and hemolytic streptococcus [15]. A rare complication of an otogenic lung abscess was reported by Saha et al., only three cases were recorded from 1957 onwards in the PubMed search [12]. Ebrahimpour et al., reports a mastoid abscess, the paper discusses how the pressure from the abscess on the bone causes necrosis of the anterior portion of the sinus resulting in the adherence of platelets and red blood cells, leading to the formation of a thrombus [14].

To treat septic venous sinus thrombosis we usually use broad‐spectrum antibiotics and surgically drain the infected site and remove the clot [14]. This was done in our case, including others in our literature review [8, 9, 10, 12, 13, 14, 15]. Shah et al. only used antibiotics due to their patient's typical manifestations and early diagnosis; symptomatic improvement was seen after a couple of weeks [14]. There are different approaches to treat sigmoid sinus thrombosis for a speedy recovery. Conducting a CT or MRI and starting the patient on broad‐spectrum intravenous antibiotics are essential. While some use mastoidectomy as the mainstay of treatment, some advocate for long‐term use of antibiotics that cross the blood brain barrier [16].

Although there is controversy regarding their use, in our case we did use anticoagulant agents. Some authors advocate their use; they say that anticoagulant treatment in patients with cerebral venous sinus thrombosis appears to be safe and is associated with an apparent reduction in the risk of death or dependency which did not reach statistical significance [21]. However, Saha et al. carried out a thrombolytic treatment in some of the cases and found a lengthening in hospital stay along with an absence of recanalization of sigmoid sinus in these patients [12]. There are risks involved in using anticoagulation therapy; it can result in release of septic emboli due to clot breakdown and hemorrhage from the surgical site [7].

## 4 Conclusion

This case underscores the critical need for early recognition and aggressive management of sigmoid and transverse sinus thrombosis as a rare yet life‐threatening complication of CSOM in children. Through timely imaging, surgical intervention, and combined antibiotic‐anticoagulant therapy, our patient achieved a favorable outcome. Clinicians should maintain a high index of suspicion for intracranial complications in pediatric patients with prolonged otologic symptoms and systemic signs of infection. Multidisciplinary collaboration and individualized care remain central to ensuring full recovery and preventing neurological sequelae.

## Author Contributions

**Abdullah Nadeem:** conceptualization, investigation, methodology, project administration, software. **Hafiza Hafsa:** data curation, investigation, validation. **Ruqaiya Shahid Raja:** conceptualization, formal analysis, writing – original draft. **Mahad Shahid Raja:** conceptualization, investigation, visualization. **Muhammad Saad Abbas:** methodology, project administration, supervision, writing – original draft, writing – review and editing. **Omer Javed Khan:** methodology, project administration, writing – original draft. **Faisal Khalique:** methodology, validation, visualization. **Riyan Imtiaz Karamat:** conceptualization, supervision. **Aymar Akilimali:** conceptualization, supervision.

## Funding

The authors have nothing to report.

## Ethics Statement

This is a case report utilizing anonymized patient information and so was classified as exempt from review from the Institutional Review Board.

## Consent

A written informed consent was obtained from the patient based on the journal's policies.

## Conflicts of Interest

The authors declare no conflicts of interest.

## Data Availability

The data that support the findings of this study are available on request from the corresponding author. The data are not publicly available due to privacy or ethical restrictions.
